# Warthin’s tumor of the larynx: a very rare case and systematic review of the literature

**DOI:** 10.1186/s40463-015-0067-5

**Published:** 2015-05-12

**Authors:** Lluís Nisa, Basile Nicolas Landis, Cinzia Salmina, Angeliki Ailianou, Eva Karamitopoulou, Roland Giger

**Affiliations:** Department of Otorhinolaryngology—Head and Neck Surgery, Inselspital, Bern University Hospital, 3010 Bern, Switzerland; Department of Clinical Research, University of Bern, 3010 Bern, Switzerland; Department of Radiation Oncology, Inselspital, Bern University Hospital, 3010 Bern, Switzerland; Department of Otorhinolaryngology—Head and Neck Surgery, Geneva University Hospital, 1211 Geneva, Switzerland; Department of Diagnostic Radiology, Geneva University Hospital, 1211 Geneva, Switzerland; Clinical Pathology Division, Institute of Pathology, University of Bern, 3010 Bern, Switzerland

**Keywords:** Warthin’s tumor, Cystadenolymphoma, Papillary cystadenoma lymphomatosum, Incidentaloma, PET/CT, Larynx

## Abstract

**Background:**

Warthin’s tumor or cystadenolymphoma (CAL) is a benign salivary gland tumor occurring almost exclusively in the parotid gland. CALs of other locations are rare.

**Case presentation:**

We report a laryngeal CAL detected in a positron emission tomography/computed tomography (PET/CT) performed for breast cancer follow-up. The tumor was successfully treated by transoral surgery.

**Discussion:**

Only 14 cases of laryngeal CAL are reported worldwide. These cases confirmed our experience of an uncomplicated and mostly successful transoral resection.

**Conclusion:**

CALs of the larynx are very rare. They are characterized by hypermetabolism in PET/CT. The increasing use of PET/CT investigations in cancer patients could give rise to more incidental findings of CALs at unusual locations such as the larynx.

## Background

Laryngeal tumors are predominantly malignant, with squamous cell carcinoma being the most frequent histopathological entity. Benign tumors represent around 5 % of all laryngeal tumors, and most of these are papillomas (more than 80 %).

Cystadenolymphoma (CAL) is a benign tumor of the salivary glands, also known as Warthin’s tumor or papillary cystadenoma lymphomatosum. CAL affects most often the parotid gland, and is the second most frequent benign salivary gland tumor following pleomorphic adenoma. CALs represent about 10 % of all salivary gland tumors, are most prevalent after the 6^th^ decade of life, have a male predominance, and are more common in smokers. In general, salivary gland tumors of the larynx are rare and malignant forms prevail. Benign salivary gland tumors of the larynx are very rare and therefore poorly documented, despite claims that oncocytic lesions occur more frequently than previously thought [1].

The aim of the present article is to report a case of supraglottic CAL and review all cases of laryngeal CALs in the literature in order to discuss presentation, histological features, management and outcome.

## Case presentation

**Fig. 1:**
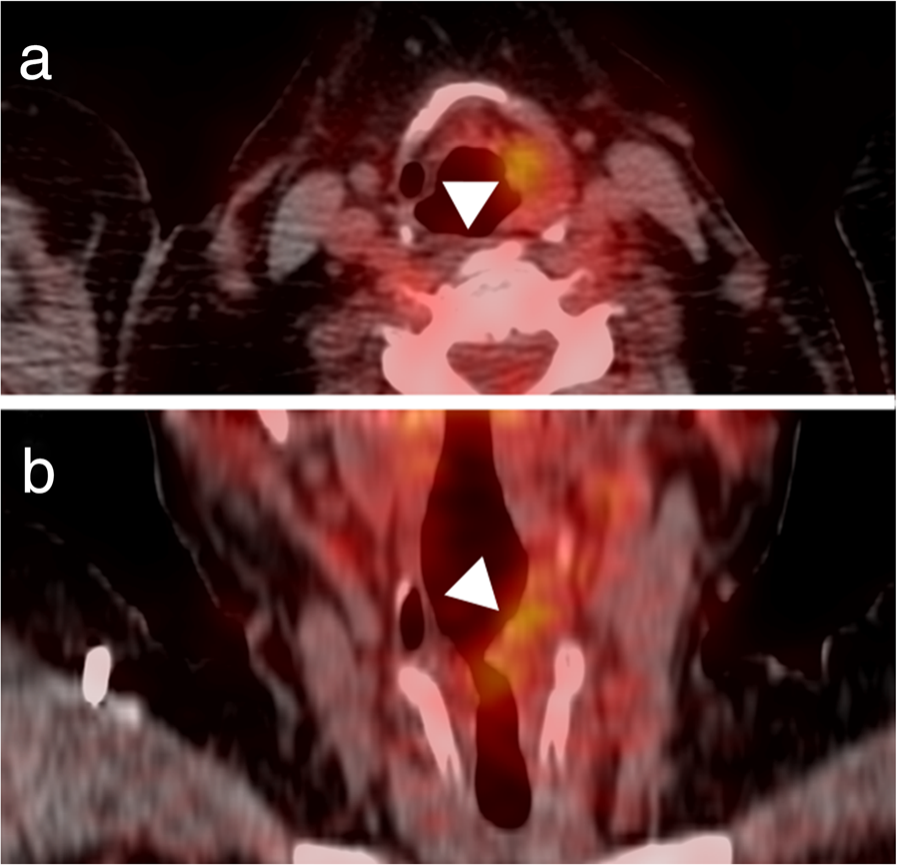
18F-FDG-PET/CT of the larynx. Intense FDG uptake in the left supraglottic region (arrowhead). **a)** axial, **b)** coronal

A 61-year-old woman was treated for a lobular carcinoma of the left breast in 2010. She had been disease-free since the end of treatment, but the 2-year follow-up 18 F-fluorodeoxyglucose positron emission tomography/computed tomography (PET/CT) showed increased uptake on the left supraglottic region (Fig. 1). With the exception of some intermittent episodes of hoarseness in the recent past, she was without symptoms. The patient is an active smoker and free of professional or environmental exposures.

**Fig. 2:**
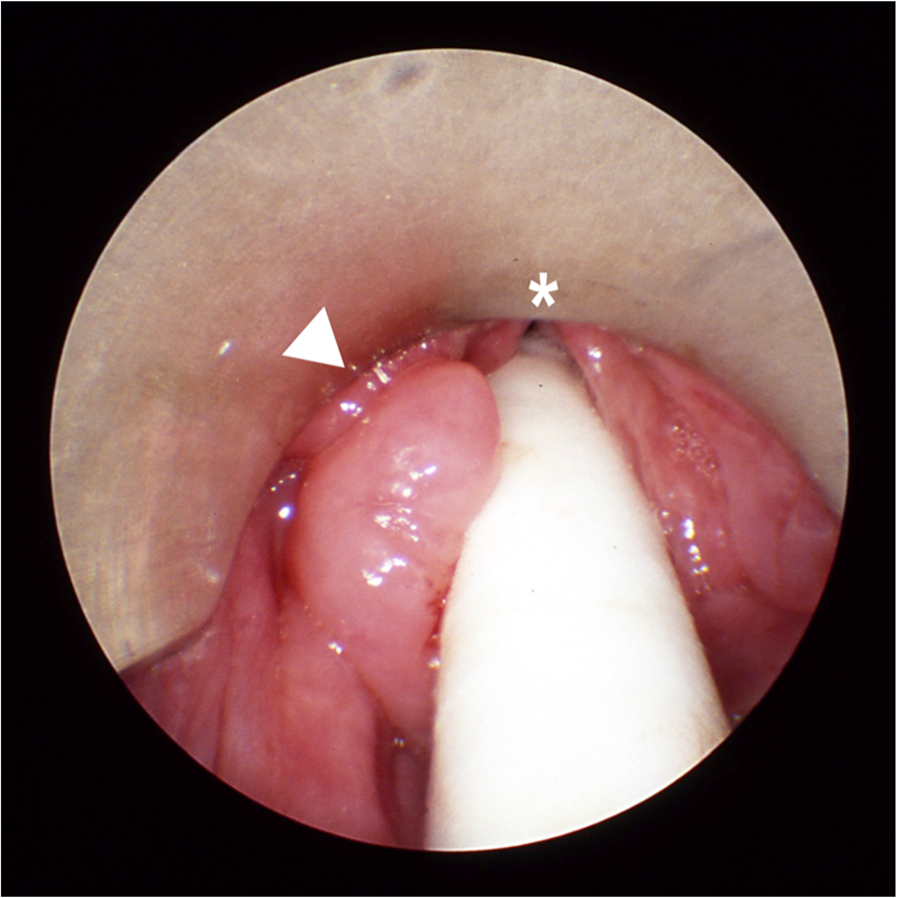
Direct laryngoscopy. A 2 × 1 cm expansive mass on the left side of the supraglottis (arrowhead; asterisk = anterior commissure)

**Fig. 3:**
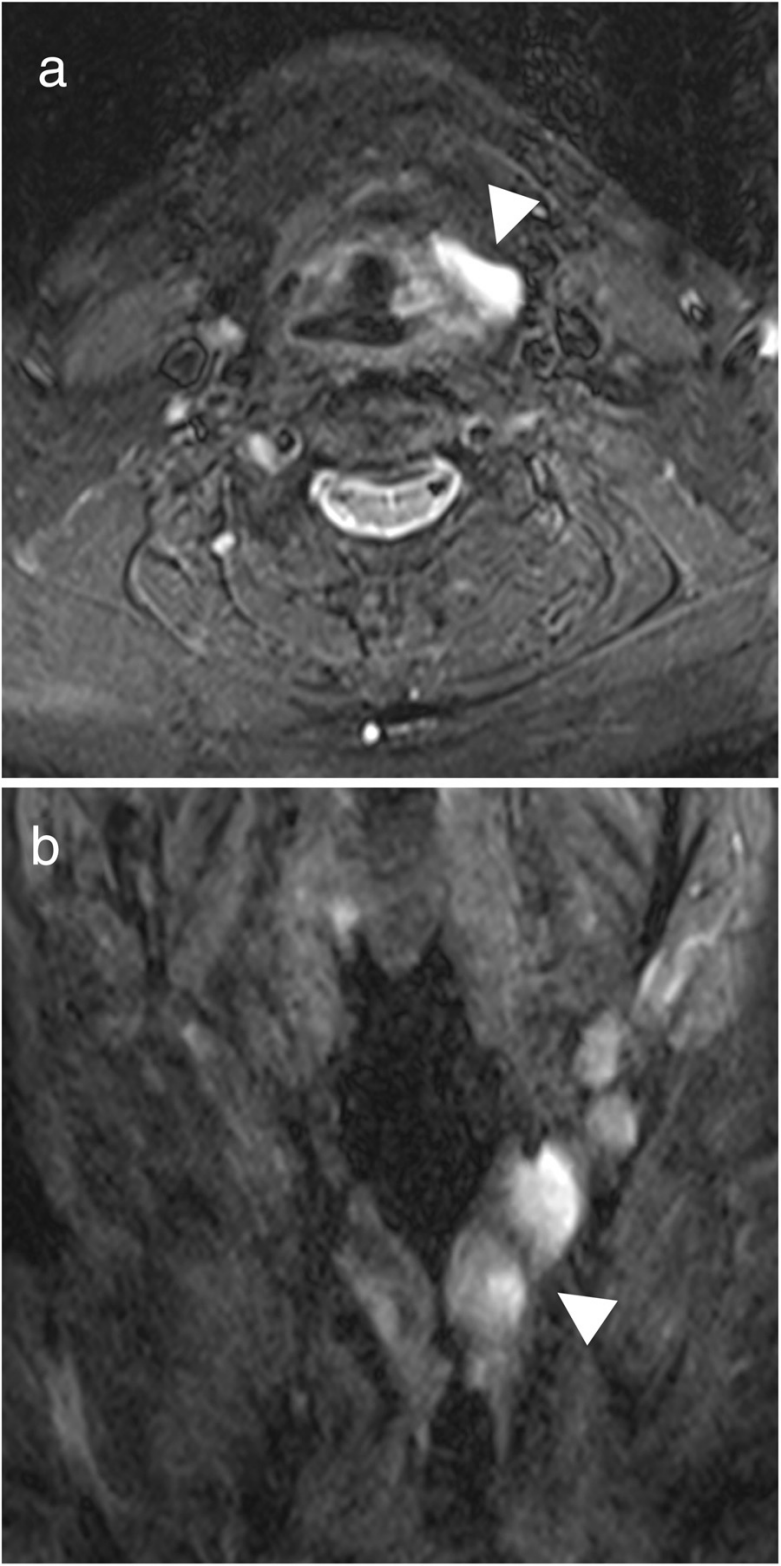
Fat-suppressed T2 weighted MRI of the larynx. Multi-cystic hyperintense lesion occupying the left supraglottic region (arrowhead). **a)** axial, **b)** coronal

**Fig. 4:**
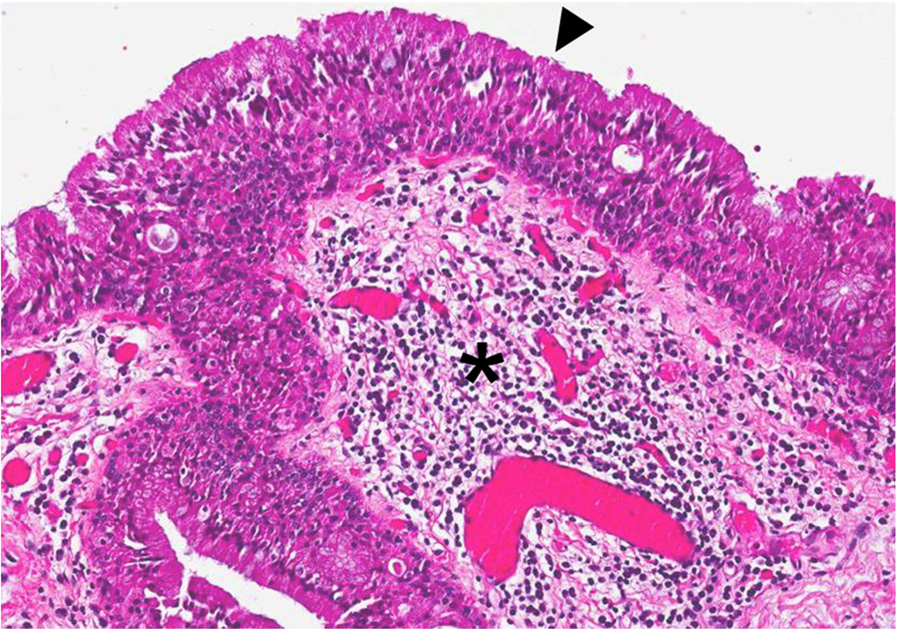
Histopathology of the laryngeal cystadenolymphoma. H&E staining of the laryngeal CAL showing oncocytic epithelium (arrowhead) surrounded by lymphoid stroma (asterisk) (magnification 40×)

Transnasal fiberoptic laryngoscopy revealed a 2 x 1 cm expansive mass involving the left side of the supraglottis. Vocal fold mobility was unaffected. A direct laryngoscopy under general anesthesia was performed (Fig. 2) for biopsy, which revealed a CAL. Complete endoscopic examination of the upper aerodigestive tract was otherwise normal. An MRI showed a 2 × 1 × 3.5 cm multi-cystic lesion of the left supraglottic region (Fig. 3). Therapeutic excision was performed endoscopically under suspension microlaryngoscopy using CO_2_ laser. Histopathologic analysis of the resection specimen confirmed the diagnosis of a CAL (Fig. 4). The follow-up was complicated by edema of the involved aryepiglottic fold causing moderate dysphagia three months after surgery. A second CO_2_ laser resection under suspension microlaryngoscopy was performed in order to resect inflammatory and edematous tissue. Further follow-up was uneventful, without any symptoms or signs of recurrence after 2 years.

## Discussion

We report a case of CAL of the supraglottic larynx identified via PET/CT done within the frame of breast cancer follow-up. Additionally, in order to obtain more relevant information on this seemingly rare entity, we performed a literature review without language restriction. Articles were searched in PubMed (up to June 2014) with the following terms: [(adenolymphoma OR lymphomatous cystadenoma OR cystadenolymphoma OR papillary cystadenoma lymphomatosum OR Warthin) AND (larynx OR laryngeal)], and with available cross-linked references from the articles found. We identified 12 eligible articles that reported 14 cases of true laryngeal CAL, histologically defined by the presence of papillary cystic epithelium and lymphoid stroma (Warthin’s tumor). Available clinical data were extracted from these articles [2-13] and summarized in Table 1. Our case was added for analysis purposes.

**Table 1:** Summary of published cases of laryngeal cystadenolymphoma

Authors	Age (years)/ Sex	Presentation	Location/Histologic features	Maximal size (cm)	Other involved sites	Surgical approach	Nbr. of operations	Outcome	Time of follow-up (months)	Comments
Heinz (1951) [2]	80/F	NR	Supraglottic	2.5	None	Transoral debulking	1	Persistence	4	-
Barbaccia et al. (1956) [3]	NR/M	Dyspnea	Supraglottic	NR	None	Tracheotomy, transcervical resection	1	FR	1	-
Tiwari and Williams (1969) [4]	72/F	Neck swelling	Supraglottic/within a laryngocele	4.5	None	Transcervical resection	1	FR	NR	-
Drut et al. (1975) [5]	70/F	Hoarseness	Supraglottic	NR	None	Transoral resection	1	FR	NR	-
Foulsham et al. (1980) [6]	66/F	Hoarseness	Supraglottic	1	None	Transoral resection	1	FR	12	Melkersson syndrome 6 years before
Engel et al. (1987) [7]	61/F	NR	NR	NR	None	Transoral resection	1	NR	NR	-
Evans et al. (1989) [8]	45/F	Hoarseness	Supraglottic	NR	None	Transoral resection	4	Recurrence, persistent supraglottic edema	NR	-
Loennecken (1989) [9]	77/F	Hoarseness Dyspnea	Supraglottic/within a laryngocele	2	Parotid gland	Transoral resection	1	Persistent supraglottic edema	NR	-
Tomik et al. (1991) [10]	72/F	Hoarseness Dyspnea	Supraglottic – glottic	NR	None	Transoral resection	1	FR	6	-
van der Wal et al. (1993) [11]	54/M	NR	NR	NR	None	NR	NR	NR	NR	-
	50/F	NR	NR	NR	NR	NR	NR	NR	NR	
	50/F	NR	NR	NR	NR	NR	NR	NR	NR	
Pätz and Möbius (1997) [12]	74/F	Dysphagia	Supraglottic	NR	Parotid gland	Transoral resection	1	FR	NR	-
Jordan et al. (1999) [13]	67/F	Hoarseness Dysphagia	Supraglottic bilaterally/within a laryngocele	3 / 2	None	NR	1	FR	NR	-
Present report (2014)	61/F	Hoarseness	Supraglottic	3.5	None	Transoral resection	2	Persistent supraglottic edema	24	-

Abbreviations: *M* – male; *F* – female; *FR –* full recovery; *NR* – not reported

Male to female ratio was 2:13, and patients’ mean age was of 64.2 ± 10.99 years (range: 45–80). The clinical presentation was not disease–but organ-specific, reflecting laryngeal involvement, with hoarseness as leading symptom in 7, dyspnea in 3, and dysphagia in 2 patients. One patient presented with neck swelling. The location was exclusively supraglottic, with glottic involvement in only one of the cases. One case showed a bilateral CAL. Two patients presented CAL within a laryngocele. Tumor size ranged between 1 and 4.5 cm. In 2 cases, synchronous CALs of the parotid gland were seen. Transoral resection was performed in 9 out of 11 patients, and 2 underwent open transcervical resection. In most cases (9 out of 11 reported) one surgical procedure was sufficient to eradicate the tumor, whereas in one case 4 operations were needed for recurrent disease and the second case showed tumoral persistence after 4 months. Our case underwent a second operation due to persistent supraglottic edema with no residual CAL during definitive histopathologic examination. Another 2 cases showed persistent laryngeal edema postoperatively.

In summary, laryngeal CAL is very rare and can most often be managed with transoral resection. Recurrence seems to be an exception in the larynx and only few complications or side effects of the treatment are reported. The unique feature of our reported case is that the patient’s CAL was detected incidentally due to regular PET/CT follow-up investigation for a previously treated breast cancer. Only retrospective inquiry showed minor laryngeal symptoms consisting of some hoarseness episodes. Accurate diagnosis requires nonetheless histopathologic examination of the resected specimens. Malignant degeneration of CALs, originating from both the epithelial and the lymphoid compartments, is a rare but possible event. In the specific case of laryngeal CALs, no case of malignant degeneration could be identified.

Workup with PET/CT has been shown to indicate false positive results in head and neck cancer patients showing a CAL within neck lymph nodes mimicking a metastasis [14]. There was similarity with our case, where FDG-uptake was found in the larynx, and initially mistaken for a breast cancer metastasis. Van der Wal et al. described the largest series of laryngeal CALs (3 cases) along with seven other cases of extraparotideal CALs (cheek, oropharynx, palate, buccal fold, lower lip, submandibular gland) [11]. As CALs show a hypermetabolism in PET/CT exams, which are increasingly performed, future PET findings might bring new insight regarding synchronous and multiple Warthin’s tumor locations and into pathogenesis of this particular tumor. While some authors have classically considered CAL to be more similar to a reactive process than to a true neoplasia, the views on pathogenesis have evolved over the last decades. Current techniques show that the epithelial portion of a substantial subset of CALs is monoclonal and frequently harbors a translocation between chromosomes 11 and 19 t (11;19), which in turn results in the fusion oncogene CRTC1-MAML2 [CREB(cAMP response element-binding protein)-regulated transcription co-activator 1–mastermind-like protein 2], which is a master key involved in several signaling pathways that contribute to cell survival and proliferation, primarily the Notch pathway [15]. Van der Wal et al. underlined the fact that laryngeal CALs are indeed rare tumors and pathologic diagnosis can be challenging, especially when it comes to distinguishing CAL from oncocytic hyperplasia or metaplasia, pointing out that the only basic difference between these entities is the lack of lymphoid stroma in oncocytic lesions [11].

Another interesting point is the potential multifocal presentation of CALs. We found 2 cases of concomitant involvement of the larynx and the parotid gland (Table 1) [9, 12]. This is not a new finding, as synchronous or metachronous development of CALs in both parotid glands is not a rare event, which raises the question if there is a need for long-term follow-up in patients with CALs in general. Although speculative in nature, we feel that careful workup in incidental PET/CT findings could reveal a previously underestimated amount of extra-salivary and multiple synchronous CAL cases. In light of the open questions regarding the pathogenesis of CALs, more cases as the present ones could bring further insight into the nature of this special benign tumor.

## Conclusion

Warthin’s tumors of the larynx are very rare. Transoral resection seems to be feasible for most cases, has little side effects, and is most often curative. As Warthin’s tumors show increased FDG-uptake similar to malignant tumors, it is hypothesized that with increasing PET/CT use, the number of Warthin’s tumor case reports outside the salivary glands may augment.

## Consent

The patient has given her consent for the Case report to be published.

## References

[1] Lundgen J, Olofsson J, Hellquest H (1928). Oncocytic lesions of the larynx. Acta Otolaryngol.

[2] Heinz I (1951). The adenolymphomata. Aust N Z J Surg.

[3] Barbaccia F, Micelli F, Tosci C (1956). Adenolinfoma della laringe. Arch Ital Otol Rhino Laringol.

[4] Tiwari RM, Williams HI (1969). Adenolymphoma in a laryngocele. J Laryngol Otol.

[5] Drut R, Di Rago C, Di Rago C (1975). Cistoadenoma papilar linfomatoso (tumor de warthin) de laringe. An Otorinolaringol Ibero Am.

[6] Foulsham CK, Snyder GG, Carpenter RJ (1981). Papillary cystadenoma lymphomatosum of the larynx. Otolaryngol Head Neck Surg.

[7] Engel P, Larsen SB, Francis D (1987). Extraparotid occurrence of papillary lymphomatous cystadenoma-Warthin’s tumor. Ugeskr Laeger.

[8] Evans RA, Cassidy MT, Russell TS (1989). Adenolymphoma of the larynx. J R Coll Surg Edinb.

[9] Loennecken I (1989). Multilokuläres vorkommen eines zystadenolymphoms (warthin-tumor) in parotis und larynx. Laryngorhinootologie.

[10] Tomik J, Papla B, Olszewski E (1991). A case of Warthin’s tumor (papillary adenolymphoma) of the larynx. Otolaryngol Pol.

[11] van der Wal JE, Davids JJ, van der Waal I (1993). Extraparotid Warthin’s tumours – report of 10 cases. Br J Oral Maxillofac Surg.

[12] Pätz S, Möbius H (1997). Simultanes auftreten eines zystadenolymphoms. HNO.

[13] Jordan J, Babiński D, Sova J (1999). Adenolymphoma (Warthin’s tumor) of the larynx: coexistence with the bilateral laryngocele. Contribution to differential diagnosis with oncocytic papillary cystadenoma. Otolaryngol Pol.

[14] Schwarz E, Hürlimann S, Soyka JD, Bortoluzzi L, Strobel K (2009). FDG-positive Warthin’s tumors in cervical lymph nodes mimicking metastases in tongue cancer staging with PET/CT. Otolaryngol Head Neck Surg.

[15] O’Neill ID (2009). New insights into the nature of Warthin’s tumour. J Oral Pathol Med.

